# Fear of negative evaluation modulates electrocortical and behavioral responses when anticipating social evaluative feedback

**DOI:** 10.3389/fnhum.2013.00936

**Published:** 2014-01-21

**Authors:** Melle J. W. Van der Molen, Eefje S. Poppelaars, Caroline T. A. Van Hartingsveldt, Anita Harrewijn, Bregtje Gunther Moor, P. Michiel Westenberg

**Affiliations:** ^1^Institute of Psychology, Faculty of Social and Behavioural Sciences, Leiden UniversityLeiden, Netherlands; ^2^Leiden Institute for Brain and CognitionLeiden, Netherlands; ^3^Department of Developmental Psychology, University of AmsterdamAmsterdam, Netherlands

**Keywords:** fear of negative evaluation, social evaluation, feedback anticipation, stimulus preceding negativity, feedback-related negativity, P3, EEG, event-related brain potentials

## Abstract

Cognitive models posit that the fear of negative evaluation (FNE) is a hallmark feature of social anxiety. As such, individuals with high FNE may show biased information processing when faced with social evaluation. The aim of the current study was to examine the neural underpinnings of anticipating and processing social-evaluative feedback, and its correlates with FNE. We used a social judgment paradigm in which female participants (*N* = 31) were asked to indicate whether they believed to be socially accepted or rejected by their peers. Anticipatory attention was indexed by the stimulus preceding negativity (SPN), while the feedback-related negativity and P3 were used to index the processing of social-evaluative feedback. Results provided evidence of an optimism bias in social peer evaluation, as participants more often predicted to be socially accepted than rejected. Participants with high levels of FNE needed more time to provide their judgments about the social-evaluative outcome. While anticipating social-evaluative feedback, SPN amplitudes were larger for anticipated social acceptance than for social rejection feedback. Interestingly, the SPN during anticipated social acceptance was larger in participants with high levels of FNE. None of the feedback-related brain potentials correlated with the FNE. Together, the results provided evidence of biased information processing in individuals with high levels of FNE when anticipating (rather than processing) social-evaluative feedback. The delayed response times in high FNE individuals were interpreted to reflect augmented vigilance imposed by the upcoming social-evaluative threat. Possibly, the SPN constitutes a neural marker of this vigilance in females with higher FNE levels, particularly when anticipating social acceptance feedback.

## INTRODUCTION

The fear of negative evaluation (FNE) is considered to be a hallmark of social anxiety. Cognitive theories posit that this fear may result from biased information processing, particularly when anticipating a fearful event (Clark and McManus, 2002). Socially anxious individuals exhibit maladaptive appraisal of social situations, which is characterized by the selective retrieval of negative information about themselves (Rapee and Heimberg, 1997). This biased information is then utilized to make negative self-evaluations (Rapee and Heimberg, 1997; Clark and McManus, 2002). Rapee and Spence (2004) proposed in their influential model that social anxiety can be viewed as lying on a continuum: the lower end of this continuum reflects a total lack of social anxiety, the middle of the continuum marks a strong desire to be positively evaluated, and the highest end of this continuum is marked by an intense fear and avoidance of social situations/interactions. Those individuals who can be placed at the highest end of this continuum meet the criteria of social anxiety disorder or social phobia (Rapee and Spence, 2004; Morrison and Heimberg, 2013). In most cognitive models it is postulated that individuals with social anxiety display a variety of information processing biases (e.g., negative self-referential biases, increased self-focused attention) that generate feelings of anxiety. This anxiety and the negative appraisal of the self contribute to the maintenance of social anxiety by a series of vicious cycles (Clark and McManus, 2002; Morrison and Heimberg, 2013). Neurocognitive theories posit that these information processing biases may be due to aberrant emotion regulation strategies, caused by impaired top-down regulation of negative affect by prefrontal brain structures (Etkin and Wager, 2007; Etkin, 2010; Brühl et al., 2011; Brühl et al., 2013). To date, however, the neural underpinnings of information processing biases related to FNE remain poorly understood. In the current study we focus on the middle end of the social anxiety continuum and will examine the neural underpinnings of social-evaluative feedback anticipation, and the processing thereof. We aim to investigate how individual differences in FNE modulate this neural activity in order to delineate the electrocortical signatures of the information processing biases implicated in social anxiety.

An appealing paradigm to study social evaluation is the social judgment paradigm introduced by Somerville et al. (2006). In this paradigm, participants are shown portrait photographs of unfamiliar peers, and are led to believe that these peers have previously formed impressions about the participant. The participant is asked to judge whether peers either formed positive (i.e., like) or negative (i.e., dislike) impressions. After each judgment, participants are provided with peer feedback that is either congruent or incongruent with their prior expectations. In this study it was shown that valence of the judgment was related to activation of the ventral anterior cingulate cortex (vACC), whereas the dorsolateral anterior cingulate cortex (dACC) was particularly sensitive to violations of participant’s expectations. In a follow up study it was demonstrated that the magnitude of the vACC activation to positive social-evaluative feedback was enhanced in individuals with low self-esteem, as compared to individuals with high self-esteem (Somerville et al., 2010). Using the same paradigm, Gunther Moor et al. (2010b) found that the magnitude of this polarization in brain activation followed a linear increase during development. This finding was accompanied by an optimistic self-evaluation bias in 19–25 years old participants. Namely, older participants made significantly more positive social evaluation judgments in comparison to younger participants. This optimistic self-evaluation bias was interpreted in terms of social belongingness theory (Baumeister and Leary, 1995), which states that social acceptance has a high evolutionary value, as it promotes survival and well-being in humans. Accordingly, it has been proposed that social-evaluative threat may serve as a signal that the need for social connection is not being satisfied. In turn, this “need to belong” may augment the desire to form bonds with other people (Maner et al., 2007). Together, the above findings suggest that people have positive expectations about social evaluation by peers, and that this optimism bias is governed by a ventral medial prefrontal neural network, brain regions frequently implicated in self-referential processing and mentalizing (Amodio and Frith, 2006). Interestingly, the magnitude of the polarization in brain activation after receiving positive vs. negative feedback seems subject to individual differences (e.g., levels of self-esteem), suggesting that the social judgment paradigm may be a suitable paradigm to examine biomarkers of social-evaluative fear, a related construct to social anxiety (Watson and Friend, 1969; Rapee and Heimberg, 1997; Weeks et al., 2005; Weeks and Howell, 2012; Levinson et al., 2013).

Due to its fine-grained temporal resolution, investigating event-related brain potentials (ERPs) could add an important dimension to our understanding of individual differences in anticipatory vs. feedback-related processing of social-evaluative information. In a recent study, Van der Veen et al. (2013) investigated feedback ERPs using the social judgment paradigm. Results of this study corroborated the enhanced brain activity after receiving social acceptance feedback. That is, participants displayed a significantly larger P3 component when they were presented with expected social acceptance feedback. However, anticipatory processes were not examined in this study, and the small sample size prohibited the authors from examining individual differences in the processing of social-evaluative feedback.

The purpose of this study was to examine individual differences in neural activity associated with the anticipation of social-evaluative feedback, as well as the processing of this information. We measured FNE in a sample of healthy female adult participants, as it was anticipated that FNE would bias both anticipatory and feedback-related neural activity, as well as behavioral judgments about the social-evaluative outcome. Although FNE only reflects a part of the social anxiety spectrum, namely the interaction anxiety subtype (Mattick and Clarke, 1998), it has been used in a host of studies as an index of non-clinical social anxiety (Wieser et al., 2009; Abraham et al., 2013; Rossignol et al., 2013; Salemink et al., 2013). We measured the stimulus preceding negativity (SPN) as a neural index of anticipatory attention. The SPN is a slow negative potential that progressively increases in amplitude prior to the onset of a feedback stimulus (Böcker and Boxtel, 1997; Brunia et al., 2011). The morphology of the SPN is dependent on the specific task parameters, but SPN amplitudes generally increase for feedback stimuli that convey affective or motivational valence (Böcker et al., 2001). Peak SPN amplitudes display a right lateralized dominance in time-estimation and gambling experiments (Brunia et al., 2011), but the anticipation of appetitive feedback stimuli (e.g., rewarding stimuli) has been associated with left-lateralized dominance of the SPN (Poli et al., 2007). Further, SPN amplitudes seem to be dependent on the level of certainty about the upcoming feedback stimulus, namely, SPN amplitudes have been found to be larger prior to unpredictable – thus uncertain – feedback stimuli (Catena et al., 2012). Since intolerance of uncertainty is posited to be a significant contributor to social-evaluative fears (Whiting et al., 2013), it was anticipated that the SPN would constitute a neural marker of this uncertainty of social evaluation. Moreover, measuring the SPN during the anticipation of both social rejection and acceptance feedback allowed us to examine whether females with high FNE would divert more attention to upcoming rejection or acceptance feedback.

The processing of social-evaluative feedback can be indexed with the feedback-related negativity and P3 components of the feedback-related brain potential. The FRN is a frontocentral negative component peaking approximately 250 ms after feedback onset, whereas the P3 shows peak amplitudes at around 300–600 ms post stimulus. The FRN is typically elicited by feedback stimuli that are incongruent with prior expectations, and is frequently interpreted to reflect performance monitoring (Van Noordt and Segalowitz, 2012). In contrast, the P3 is considered to be a more cognitive component, governed by top-down attentional control mechanisms (Polich, 2007). A recent study revealed that P3 amplitudes in the social judgment paradigm were larger for positive than for negative feedback (Van der Veen et al., 2013), a finding that was interpreted to reflect a confirmation of social acceptance and its inherent feeling of reward.

In the current study we examined the following hypotheses: (1) conform findings of Gunther Moor et al. (2010b) and in line with Social Belongingness Theory (Baumeister and Leary, 1995), we hypothesized an optimism bias in our participant sample – such that participants would anticipate social acceptance more often than social rejection; (2) Based on the notion that socially anxious individuals anticipate social rejection more often (Clark and McManus, 2002), it was anticipated that this optimistic self-evaluation bias would only be present in females with low FNE levels; (3) In line with the notion of the uncertainty hypothesis of the SPN (Catena et al., 2012), we expected that SPN amplitudes would be larger for social acceptance than for rejection judgments in females with higher FNE levels, since females high in FNE may expect rejection more often, rendering social acceptance more unlikely; (4) Based on fMRI results showing increased brain activation after receiving positive-evaluative feedback (Somerville et al., 2006; Gunther Moor et al., 2010b; Somerville et al., 2010), we anticipated larger P3 amplitudes when feedback communicated social acceptance. It was hypothesized that this effect would be more pronounced in females with higher FNE levels, since P3 amplitude is modulated both by valence and expectancy (Ferdinand et al., 2012). As we anticipated that females high in FNE would predict social acceptance less often, feedback signaling acceptance would be more surprising and thus render larger P3 amplitudes.

## MATERIALS AND METHODS

### PARTICIPANTS

Thirty-one female participants aged between 18 and 24 years (mean age = 19.78; SD = 1.45) participated in this study. All participants were right handed as verified with the Edinburgh Handedness Inventory (Oldfield, 1971) and had no history of neurological or psychiatric disorders. Participants had normal or corrected-to-normal vision, and were free from psychoactive medication. Participants were recruited from or within the proximity of the university, provided signed informed consent, and were awarded course credit or fixed payment for their participation. None of the participants had any doubts about the cover story (see Experimental design and procedure). The protocol for this study was reviewed and approved by the medical ethical review committee of the Leiden University Medical Center.

### FEAR OF NEGATIVE EVALUATION

Fear of negative evaluation was assessed with the Dutch translated brief version of the *Fear of Negative Evaluation Scale, revised* (BFNE-R; Bögels and Reith, 1999; Carleton et al., 2006). The BFNE-R has demonstrated excellent levels of internal consistency and test–retest reliability, correlates highly with the full scale FNE, and is a commonly used measure of social anxiety (Collins et al., 2005; Carleton et al., 2011). The BFNE-R consists of 12 statements about social-evaluative situations. Participants have to indicate on a 5-point Likert scale the degree to which each statement applies to them (0 = not at all characteristic of me; 4 = extremely characteristic of me). Carleton et al. (2011) showed that a cut-off score of 38 can be employed to specify individuals showing clinical signs of social anxiety disorder. An excellent internal consistency was obtained for the items within the current sample (α = 0.95). To test the validity of the FNE scores, we measured levels of social anxiety, self-esteem, behavioral inhibition, and rejection sensitivity, with the Liebowitz Social Anxiety Scale (LSAS; Liebowitz, 1987), the Rosenberg Self-Esteem Scale (RSES; Rosenberg, 1965), Behavioral Inhibition Scale (BIS; Carver and White, 1994), and Rejection Sensitivity Questionnaire (RSQ; Downey and Feldman, 1996), respectively. Mean scores on the self-report measures are presented in **Table 1**. The FNE correlated significantly with all measures and yielded good-to-excellent internal consistencies (see **Table 2**).

**Table 1:** Means, standard deviations (SD) and range (minimum– maximum) of the scores on the self-reported questionnaires.

Questionnaire	Mean (SD)	Range (min.–max.)
Fear of Negative Evaluation (FNE)	23.00 (11.35)	4–47
Social Anxiety (LSAS)	35.48 (15.33)	12–87
Self-Esteem (RSES)	9.77 (4.91)	1–18
Rejection Sensitivity (RSS)	7.82 (3.81)	2.78–14.61
Behavioral Inhibition (BIS)	22.26 (3.45)	16–27

**Table 2:** bf Internal consistencies of the questionnaires used to index social anxiety, self-esteem, rejection sensitivity, and behavioral inhibition.

Questionnaire	Cronbach’s alpha	Correlation with FNE
Social Anxiety (LSAS)	0.91	*r*(31) = 0.36, *p* = 0.045
Self-Esteem (RSES)	0.87	*r*(31) = 0.63, *p* < 0.0001
Rejection Sensitivity (RSS)	0.88	*r*(31) = 0.59, *p* = 0.001
Behavioral inhibition (BIS)	0.86	*r*(31) = 0.56, *p* = 0.001

*Scores on these measures correlated positively with scores on the fear of negative evaluation (FNE) questionnaire.*

### EXPERIMENTAL DESIGN AND PROCEDURE

A modified version of the social judgment paradigm was used (Somerville et al., 2006; Gunther Moor et al., 2010b). With a cover story, participants were led to believe that they would participate in a study on first impressions. Approximately 2 weeks prior to the experimental session, participants were asked to send a personal portrait photograph to the investigators. A panel of peer undergraduate students from other universities would evaluate this photograph, and provide a judgment based on their first impressions (i.e., like or dislike the person on the photograph). At the day of the experiment, participants completed the social judgment paradigm together with another cognitive task (order of presentation was counterbalanced between participants) of which the data will not be presented in this study. Prior to the social judgment experiment, participants were told that they would see portrait photographs of each member of this panel of peers. Their task was to judge whether this peer member liked or disliked the participant. A total of 160 photographs of peers were used (50% male), derived from taking photographs of undergraduates from different universities. Photos of the peers were presented at a 17-inch monitor [60 Hz refresh rate; visual angle (width/height) = (4.66°× 6.05°)] using E-prime 2.0 stimulus presentation software (Psychology Software Tools, Pittsburgh, PA, USA). All peer photographs had a neutral facial expression, as ascertained with the Self-Assessment Manikin (SAM) on a 9-point scale (Bradley and Lang, 1994). These SAM-ratings of arousal and valence were obtained from an independent sample of volunteers (*N* = 21), gender and age-matched to the participants.

**FIGURE 1:**
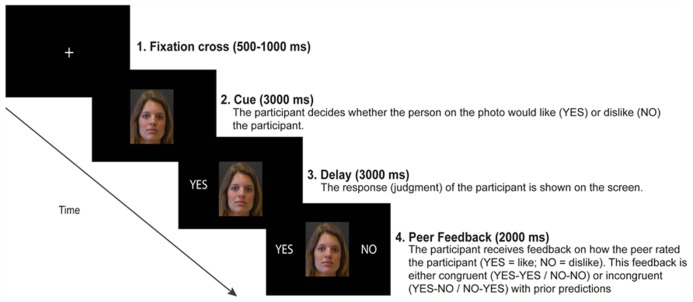
**An illustration of a single trial in the social evaluation paradigm. ** On each trial, participants are presented with a photograph of a peer. The participant is asked to judge whether the peer would either like (accept) or dislike (reject) the participant. Based on judgment type (“YES” or “NO”) and feedback type (“YES” or “NO”), four possible feedback conditions were included: expected social acceptance (“YES–YES”), expected social rejection (“NO–NO”), unexpected social acceptance (“NO–YES”), or unexpected social rejection (“YES–NO”). This particular trial shows an example of unexpected social rejection.

An illustration of a single trial is shown in **Figure 1**. Each trial commenced with the depiction of the cue for 3000 ms displaying the neutral face of the peer. The cue remained on the screen until the end of the trial. During this 3000 ms interval, participants were required to provide their positive (i.e., “acceptance”) or negative (i.e., “rejection”) judgments by pressing one of two buttons on an armrest. The order of which button (left or right) corresponded with acceptance (“YES”) or rejection (“NO”) anticipations was counterbalanced between participants. If participants did not respond within 3000 ms after onset of the cue, the message “too slow” appeared on the screen signaling the end of the trial. Participants were instructed that they had about 3000 ms to provide their judgment. Participants were told that they did not have to respond as fast as possible, but rather they had to seriously evaluate whether the person on the photograph liked or disliked the participant. Trials on which participants responded too slow (i.e., after 3000 ms from cue onset) were excluded from the analysis. When participants provided their judgment, a visualization of their response (“YES” or “NO”) was immediately displayed to the left of the peer’s face. After a fixed delay of 3000 ms (i.e., the anticipation period), feedback appeared to the right of the peer’s face for 2000 ms, communicating either social acceptance (“YES”) or rejection (“NO”). Social rejection feedback (“NO”) was presented on 50% of the trials^1^. Feedback in this paradigm was not actual peer-feedback, but fictitious feedback that was pseudo-randomly generated by the computer, such that at least on 50% of the trials participants received acceptance feedback. Between trials, a fixation cross was presented in the center of the screen with a jittered duration between 500 and 1500 ms. Participants started with 10 practice trials, and then completed three experimental blocks comprising 50 trials each. At the end of the experiment, electroencephalography (EEG) equipment was detached and participants were asked to write down their experiences and thoughts about the experiment. Subsequently, participants filled out the abovementioned questionnaires. None of the participants had doubts about the cover story. Participants were debriefed about the true purpose of this study by letter after the last participant was examined.

### BEHAVIORAL ANALYSIS

The following behavioral data was used for analysis: the number of acceptance and rejection judgments, as well as the reaction time (RT) that was needed to provide these judgments. A bias score was calculated to examine whether participants anticipated significantly more acceptance than rejection feedback (Van der Veen et al., 2013). This bias score was derived from dividing the number of acceptance judgments by the number of total judgments. This bias score either reflects an optimism bias (>50%) or a pessimism bias (<50%).

### EEG RECORDING AND SIGNAL PROCESSING

Electroencephalography time series were recorded at a 1024 Hz sampling rate from 64 Ag–AgCl electrodes mounted in an elastic electrode cap (10/20 placement) using a BioSemi Active Two system (Biosemi, Amsterdam, The Netherlands). The BioSemi system replaces the ground electrode by a feedback loop consisting of the common mode sense (CMS) electrode and Driven right leg (DRL) electrode; CMS was used as the online reference. Horizontal electrooculography (HEOG) was measured from two electrodes placed at the left and right canthus; vertical EOG (VEOG) was measured from two electrodes placed above and below the left eye. Two electrodes were placed at the mastoids. Offline processing of the EEG was performed with Brain Vision Analyzer 2 (Brain Products GmbH). The EEG signal was down-sampled to 512 Hz, re-referenced to the average of the left and right mastoids and offline band-pass filtered between 0.1 and 40 Hz (24 dB/oct), with a 50 Hz notch filter. Ocular artifacts were removed automatically using the Infomax Ocular ICA method as implemented in Brain Vision Analyzer. Subsequently, segments were created to isolate the SPN and feedback-related brain potentials (FRN and P3). All segments were visually inspected for remaining artifacts. The average number of segments used for analyses of the SPN and feedback-related components is presented in **Table 3**.

**Table 3:** bf Means, standard deviations (SD), and range (minimum– maximum) of the number of trials that were used to calculate the SPN and the feedback-related brain potentials.

Component (condition)	Mean (SD)	Range (min.–max.)
SPN (predicted acceptance)	74.42 (13.32)	44–104
SPN (predicted rejection)	59.98 (12.63)	29–90
Feedback (expected acceptance)	36.98 (7.09)	20–55
Feedback (unexpected rejection)	37.22 (8.75)	19–57
Feedback (expected rejection)	29.98 (8.22)	13–49
Feedback (unexpected acceptance)	30.02 (6.64)	14–46

To isolate the SPN, 3500 ms segments were created including a 200 ms post feedback-stimulus interval. These segments included the participants’ judgments (responses) occurring at 3000 ms prior to the onset of the feedback stimulus. The 2400–2000 ms interval was used for baseline correction, as this time period most likely is the start of the anticipation period and visual inspection verified the absence of any residual motor activity. Previous studies have shown that setting baseline corrections prior to motor responses may confound the SPN by including anticipatory activity associated with the response preparation (Brunia et al., 2011). In line with prior studies, the SPN was calculated using a mean amplitude measurement within the 200 ms interval prior to the onset of the feedback stimulus at the Fz electrode (Kotani and Aihara, 1999; Ohgami et al., 2006; Stavropoulos and Carver, 2013). Although the SPN usually reaches peak amplitude at frontal electrode sites (Böcker et al., 2001; Brunia et al., 2011) data from the parietal–occipital electrodes PO7 and PO8 were analyzed also, as visual inspection of the data revealed that the SPN reached largest amplitudes over these leads in both anticipation conditions^2^. 

**FIGURE 2:**
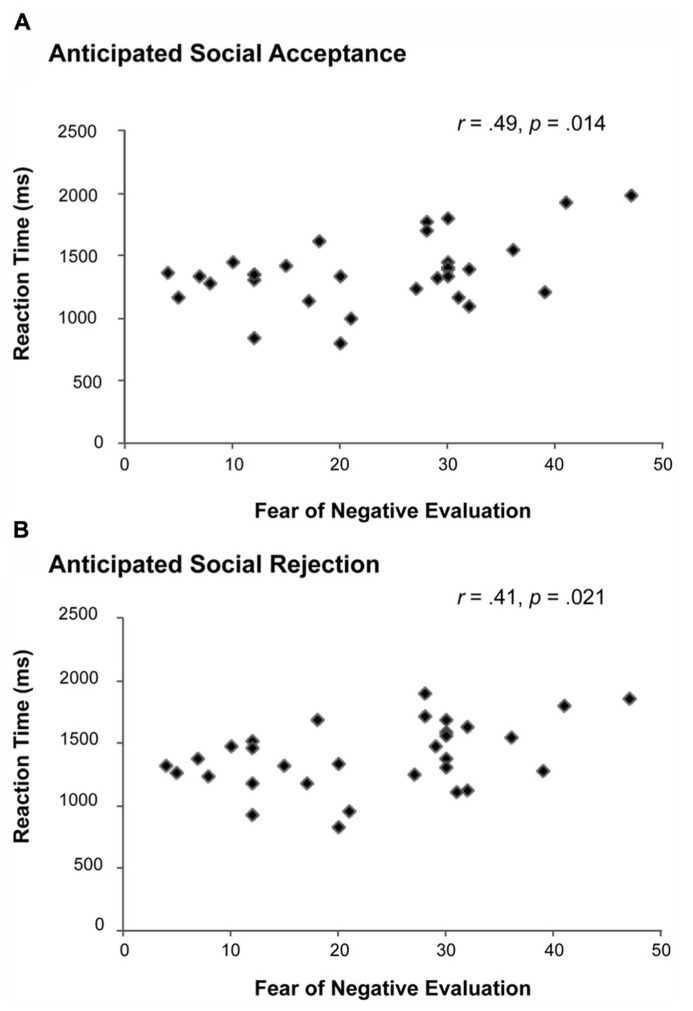
**Correlations between the Fear of Negative Evaluation (FNE) and the RT of social acceptance (A) and social rejection (B) judgments.** Individuals with higher levels of FNE needed significantly more time to provide both social acceptance (“YES”) and social rejection (“NO”) judgments.

To isolate the feedback-related ERPs, 1200 ms epochs were created including a 200 ms pre-stimulus interval, which was used for baseline correction. FRN amplitude was measured using the peak-to-peak method described by Holroyd et al. (2003), since a single peak measurement often confounds FRN amplitude due to overlap with the earlier P2 component (Luck, 2005). In line with Holroyd et al. (2003), the onset of the FRN was determined by finding the most positive peak within a 200–300 ms time window (i.e., the P2 component). From the onset of the negativity, the most negative peak was determined within the 250–350 ms time window. FRN amplitudes were obtained by subtracting the P2 peak from this most negative value. Finally, the feedback-related P3 component was examined by calculating the mean amplitude in a time window between 360 and 440 ms, as recommended by Luck (2005).

### STATISTICAL ANALYSES

Statistical Analyses were performed in three successive steps: (1) Task performance was analyzed using a one-sample *t*-test to verify a significant difference in judgment type. Pearson product-moment correlation was performed to examine the correlation between judgment type and level of FNE; (2) Anticipatory brain activity (SPN) was assessed using a repeated measures ANOVA with Site (three levels: Fz, PO7, PO8) and Judgment (two levels: acceptance, rejection) as within-subject factors to test where the SPN reached peak amplitude, and whether this differed between Judgment types. *Post hoc* analyses were performed to explore significant main or interaction effects; (3) For the feedback-related ERPs, a repeated measures analysis was performed, separately for the FRN and P3, with the within-subject factors Congruency (two levels: congruent, incongruent) and Valence (two levels: positive, negative). *Post hoc* analyses were performed to explore significant main or interaction effects. Pearson product-moment correlation analyses were performed to test our behavioral and ERP hypotheses. Bonferroni corrections for multiple comparisons were applied. Statistical analyses were performed using IBM SPSS Statistics 19 (IBM Corporation, 2010). The behavioral and EEG data were inspected for outliers (i.e., data points above or below two standard deviations of the sample’s mean). No outliers were detected. Alpha was set at 0.05 and additional *post hoc* significance testing was performed using Bonferroni correction for multiple comparisons. Greenhouse–Geisser correction was applied when necessary, and non-adjusted degrees of freedom were reported for transparency^3^.

## RESULTS

### TASK PERFORMANCE

An average response bias score of 56% (SD = 0.09) was observed indicating that participants displayed an optimism bias in anticipating more social acceptance feedback. A one-sample *t*-test verified that this bias score differed significantly from the baseline (i.e., 50%), as participants anticipated acceptance feedback (Mean number of trials = 82.42, SD = 13.76) more often than rejection feedback (Mean number of trials = 65.68, SD = 14.17), *t*(30) = 3.36, *p* = 0.002. Next, we tested the hypothesis that females with higher levels of FNE would anticipate rejection feedback more often, however, no significant positive correlation yielded between FNE scores and the percentage of negative judgments, *r*(31) = 0.30, *p* = 0.106.

Subsequently, we analyzed the RT data of the anticipated acceptance and rejection judgments using a one-sample *t*-test. No significant differences were observed in the RT of acceptance (Mean RT = 1366.91, SD = 274.85) and rejection judgments (Mean RT = 1391.34, SD = 266.66). However, as shown in **Figure 2**, a Pearson product-moment correlation analysis revealed that females with higher FNE-S scores displayed longer RTs for predicting acceptance, *r*(31) = 0.44, *p* = 0.014, and rejection feedback, *r*(31) = 0.41, *p* = 0.021. This response time effect remained significant after controlling for the effect of behavioral inhibition for predicting acceptance (*p* = 0.013) and rejection feedback (*p* = 0.022), respectively.

### ANTICIPATORY BRAIN ACTIVITY: STIMULUS PRECEDING NEGATIVITY

**FIGURE 3:**
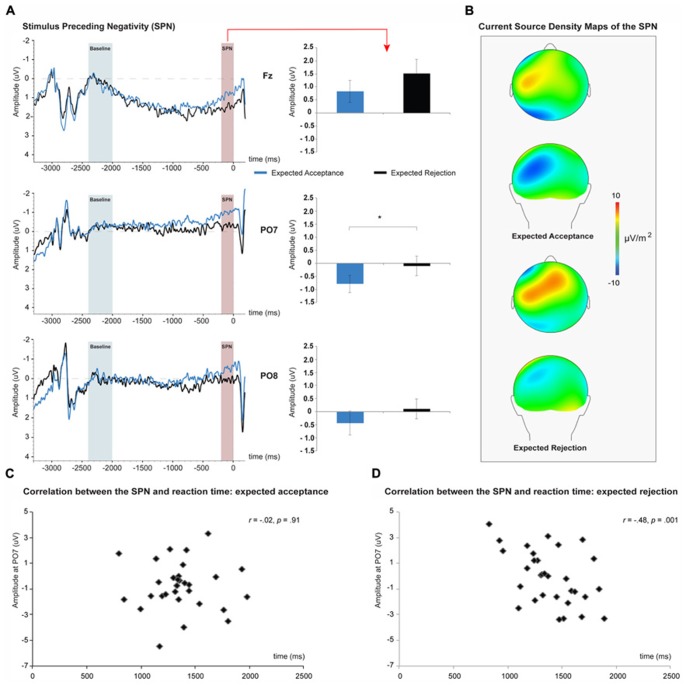
**The stimulus preceding negativity (SPN) associated with social-evaluative feedback anticipation.** SPN amplitudes were larger for expected social acceptance than for social rejection, and reached peak amplitudes at PO7 **(A)**. Current source density maps show the left posterior dominance of the SPN **(B)**. Correlations between SPN amplitude and reaction time (RT) of the judgments for expected social acceptance **(C)** and social rejection **(D)** feedback. An increase in SPN amplitude during expected social rejection was associated with a significant increase in RT of the corresponding judgment. *p < 0.05.

Results of average SPN amplitudes per judgment type are depicted in **Figure 3** for the Fz, PO7, and PO8 electrodes. Peak SPN amplitudes within the 200 ms time-window before the onset of the feedback stimulus were extracted from the Fz, PO7, and PO8 electrodes (see **Figure 3**). As revealed by a main effect of Site, SPN peak amplitude was larger at PO7 than at Fz and PO8, *F*(1,30) = 9.16, *p* = 0.005, *η p*^2^ = 0.23. The main effect of Judgment revealed that the SPN was more negative for acceptance than for rejection judgments, *F*(1,30) = 6.21, *p* = 0.018, *η p*^2^ = 0.23. No significant interaction between site and judgment was observed, *F*(2,60) = 0.05, *p* = 0.950, *η p*^2^ = 0.00. Pearson product-moment correlation analyses revealed a significant correlation between the RT for anticipated social rejection judgments and the corresponding SPN, *r*(31) = -0.48, *p* = 0.001 (see **Figure 3**).

### CORRELATIONS BETWEEN THE SPN AND FNE

**FIGURE 4:**
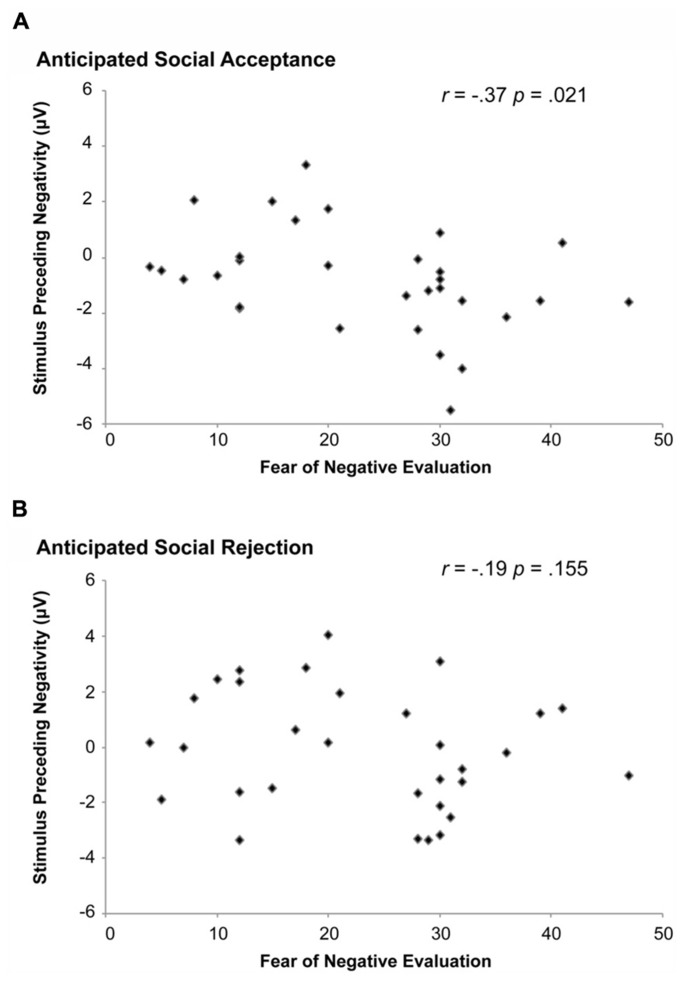
**Correlation between SPN amplitude and the fear of negative evaluation (FNE).** A significant correlation was observed between the SPN and FNE when participants were anticipating social acceptance **(A)**. This effect was not observed when participants were anticipating social rejection **(B)**.

A subsequent step was to examine whether SPN amplitudes during anticipated social acceptance or rejection could predict the level of FNE, as indexed with the FNE. Pearson correlations were run between the SPN amplitudes during positive and negative feedback anticipation, respectively, with the FNE scores. SPN amplitudes at the PO7 were used, since SPN amplitudes were largest at this lead. As shown in **Figure 4**, SPN amplitudes associated with acceptance judgments correlated significantly with FNE-S scores, *r*(31) = -0.37, *p* = 0.021. No significant correlation was found between the SPN associated with anticipated social rejection and FNE-S scores.

### FEEDBACK RELATED BRAIN ACTIVITY: FRN AND P3

**FIGURE 5:**
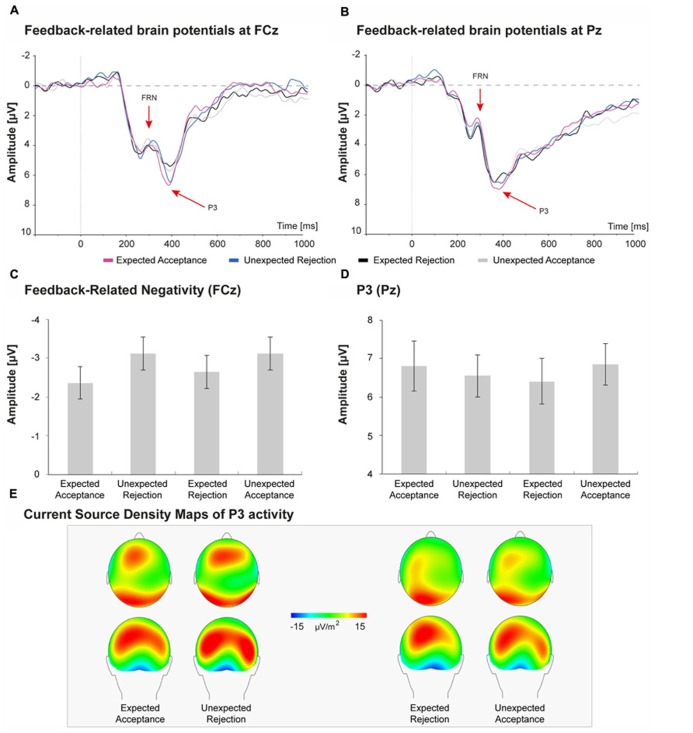
**Feedback-related brain potentials elicited by social acceptance and rejection feedback (A,B).** FRN amplitudes were largest at FCz **(A)**, whereas the P3 reached peak amplitudes at Pz **(B)**. **C,D** shows peak amplitudes of the FRN and P3 for the four social-evaluative feedback conditions. The FRN seems sensitive to congruency of feedback; however, this effect was not significant. The P3 seems larger for social acceptance feedback than for social rejection feedback, but also these differences were not significant. **E** displays the current sources density maps of P3 activity.

Brain potentials elicited by the feedback stimuli are depicted in **Figure 5**. Peak FRN amplitudes at electrode FCz were submitted to a repeated measures analysis with Congruency (two levels: congruent, incongruent) and Valence (two levels: positive, negative) as within-subject factors. The main effect of congruency revealed that FRN amplitudes for incongruent feedback were larger than for congruent feedback, however, this difference just failed to reach levels of significance, *F*(1,30) = 3.84, *p* = 0.059, *η p*^2^ = 0.11. The main effect of valence was also not significant, *F*(1,30) = 1.04, *p* = 0.317, *η p*^2^ = 0.03, suggesting that FRN amplitude did not differ between positive and negative feedback. No significant interaction between Congruency and Valence was observed, *F*(2,60) = 0.00, *p* = 0.953, *η p*^2^ = 0.00.

Peak P3 amplitudes at Pz were submitted to a repeated measures analysis with Congruency (two levels: congruent, incongruent) and Valence (two levels: positive, negative) as within-subject factors. As expected, P3 amplitudes for unexpected feedback did not differ from expected feedback, *F*(1,30) = 0.08, *p* = 0.783, *η p*^2^ = 0.00. In contrast with our expectations, the main effect of Valence was also not significant, *F*(1,30) = 1.50, *p* = 0.230, *η p*^2^ = 0.05, suggesting that P3 amplitude was not significantly larger for positive than for negative social-evaluative feedback. No significant interaction between Congruency and Valence was observed for the P3, *F*(2,60) = 0.04, *p* = 0.849, *η p*^2^ = 0.03.

### CORRELATIONS BETWEEN THE SPN AND FEEDBACK-RELATED BRAIN POTENTIALS

Next, Pearson product-moment correlations were run between the SPN and feedback-related brain potentials. We examined these correlations between SPN and the feedback components separately for anticipated acceptance and anticipated rejection. This resulted in two sets of eight correlations for (1) the SPN during anticipated acceptance versus the FRN and P3 in the four conditions (i.e., expected acceptance/rejection, unexpected acceptance/rejection), and (2) the SPN during anticipated rejection versus the FRN and P3 in the four conditions (i.e., expected acceptance/rejection, unexpected acceptance/rejection). Bonferroni correction for multiple comparisons was used (i.e., *p *< 0.006). Results revealed that an increase in SPN amplitude in the anticipated acceptance condition (“Yes” anticipations) was associated with a significant increase in P3 amplitude after anticipated acceptance (“Yes” feedback), *r*(31) = -0.47, *p* = 0.004. Partial correlation analysis revealed that this effect did not remain significant after controlling for levels of FNE, *r*(31) = 0.42, *p* = 0.021.

### CORRELATIONS BETWEEN FEEDBACK-RELATED BRAIN ACTIVITY AND FNE

Lastly, we examined whether feedback-related brain activity correlated with FNE. However, no significant results were observed (all *p*’s > 0.006).

## DISCUSSION

The impetus of this study was to investigate precursors of fear of social evaluation by examining behavioral and electrophysiological correlates of social-evaluative feedback anticipation and processing. We used a social-judgment paradigm in which participants were asked to indicate whether they believed to be accepted or rejected by their peers. In line with our hypothesis, results provided evidence of an optimism bias in social peer evaluation; namely, participants more often predicted to be socially accepted than rejected by peers. We did not find evidence for our hypothesis that the number of social rejection judgments correlated positively with the level of FNE in female participants. Interestingly, however, the current study shows that an increase in FNE levels corresponded with a significant increase in the response time of the participants to provide their judgments about upcoming social evaluation. The SPN – a brain potential associated with feedback anticipation – was larger during anticipated acceptance than rejection feedback. Furthermore, SPN amplitudes correlated positively with the level of FNE when participants were anticipating social acceptance feedback. Together, the current study provides evidence of information processing biases during social-evaluative feedback anticipation in adult females, which are modulated by the level of FNE.

In line with our hypothesis, we observed a significantly larger proportion of acceptance judgments compared to rejection judgments. This corroborates previous findings of two studies by Gunther Moor et al. (2010a, b), and may be indicative of an optimistic self-evaluation bias. Hepper et al. (2011) recently demonstrated that expectations about social-evaluative feedback are generally positive, a finding that was interpreted to reflect the situational motivation to self-enhance. According to self-enhancement theory, people have the tendency to see themselves better than they actually are (Taylor and Brown, 1988). Thus, people may anticipate more positive than negative self-evaluations. This optimistic self-evaluation bias is also in accordance with social belongingness theory (Baumeister and Leary, 1995), which states that people have a fundamental need for positive social relationships. This desire of social belongingness is an evolutionary-rooted human motivation to form and maintain social bonds, since these social bonds increase the chances for socio-emotional and physical well-being (Macdonald and Leary, 2005). The strength of this “need to belong” has furthermore been demonstrated by Maner et al. (2007), who observed that even the experience of social exclusion elicited the desire to form social bonds with other people and allocate positive evaluations to others, in the hope to establish renewed social connections.

In the current study, we examined whether this optimistic self-evaluation bias was related to levels of FNE as well as levels of generalized social anxiety (LSAS), but we found no evidence for such a correlation. We did find that participants with higher levels in FNE were significantly slower in judging whether social-evaluative feedback was positive or negative. This increment in response time in those females higher in FNE may be due to increased self-focused attention and vigilance imposed by the task demands, which could subsequently compromise information processing efficiency. In the current study, the social-evaluative threat may have prompted an increase in self-focused attention – a stimulus-driven process that is posited to interfere with disengaging attention from socially threatening stimuli – fueling maladaptive cognitions, and resulting in a greater effort in preparing responses (Judah et al., 2013). Although speculative, mainly due to the absence of an objective measure of self-focused attention in the current study, this notion is in line with the attentional control theory (ACT), which states that anxiety impairs processing efficiency in conditions that place a high demand on cognitive resources (Eysenck et al., 2007).

At the electrocortical level, we observed that SPN amplitudes were significantly larger when participants were anticipating social acceptance compared to social rejection feedback. The left parietal–occipital predominance of the SPN was most likely due to the switch of attention to the contralateral visual field, since feedback stimuli were presented right from the photographs of peers. The functional significance of the SPN is often debated, but there is accumulating evidence suggesting that the SPN reflects affective motivational anticipatory processes (Poli et al., 2007; Brunia et al., 2011). A host of electrophysiological studies revealed that SPN amplitudes tend to be larger for stimuli that are rewarding (Ohgami et al., 2004; Mattox et al., 2006; Ohgami et al., 2006; Stavropoulos and Carver, 2013). In the current study, receiving social acceptance feedback would be more rewarding than rejection feedback, which could be related to the enhanced SPN amplitudes when participants were anticipating social acceptance. This interpretation meshes with the aforementioned social belongingness theory, namely that participants were anticipating social acceptance more often than rejection, as social acceptance facilitates the formation of new social bonds and general wellbeing of the individual.

An alternative account on the functionality of the SPN is the “uncertainty hypothesis”, which posits that the SPN would be larger when predictions are made for highly unpredictable or uncertain outcomes (Catena et al., 2012). In the current study we observed that SPN amplitudes were larger in participants who were slower in providing their judgment about upcoming social rejection feedback. This slowing in response time for predicting social rejection feedback may be indicative of uncertainty about social rejection, as the optimistic self-evaluation bias revealed that participants more often predicted to be socially accepted. However, this interpretation is in stark contrast to the observation that SPN amplitudes were larger when participants anticipated to be socially accepted. Moreover, results showed that SPN amplitudes correlated positively with the levels of FNE when participants anticipated social acceptance. Based on the uncertainty hypothesis it was a priori expected that females with higher levels of FNE would anticipate social rejection more often, thereby rendering social acceptance as less likely and therefore uncertain. Due to the absence of a pessimistic self-evaluation bias in females with higher levels of FNE, it seems unlikely that the augmented SPN in high FNE females can be explained by uncertainty about the social-evaluative outcome. The larger SPN amplitudes when anticipating social acceptance feedback may be reflective of the intrinsic motivation to be socially accepted, which dovetails with the aforementioned social belongingness theory (Baumeister and Leary, 1995). Also, the distribution of FNE scores in the current sample indicated that only a few participants (*N* = 3) met the criteria for higher levels of social anxiety (Carleton et al., 2011), whereas the majority of the participants could be placed on the middle range of the social anxiety continuum (Rapee and Spence, 2004). According to Rapee and Spence (2004), these individuals can often be characterized by having a strong desire to be positively evaluated. Future studies should examine whether the behavioral and electrocortical findings will be exaggerated in participants with clinical levels of social anxiety, or whether these participants will (1) anticipate rejection feedback more often, and (2) will display a differential pattern of brain activation during social-evaluative feedback anticipation.

The processing of social-evaluative feedback was indexed by the FRN and P3. Results revealed that feedback that violated prior anticipations (e.g., unexpected acceptance and unexpected rejection) was associated with larger FRN amplitudes, relative to feedback that was congruent with prior anticipations; however, this incongruency effect just failed to reach levels of significance. The FRN is typically seen after incongruent feedback communicating unexpected feedback or poor performance (Van Noordt and Segalowitz, 2012). Although social-evaluative feedback in the current study could be incongruent with prior expectations, the absence of a significant incongruency effect may be explained by the fact that incongruent feedback did not communicate task performance. That is, FRN amplitudes may be larger for incongruent feedback that can be used to optimize future task performance. Based on prior neuroimaging findings of Somerville et al. (2006), we anticipated a pronounced FRN in this study, since a candidate source of the FRN (i.e., the ACC) seems differentially activated by social-evaluative feedback and expectancy violations. These authors found that the dorsal ACC was particularly activated by incongruent feedback, whereas the vACC was activated by acceptance feedback. The surface EEG potentials in the current study evidently lacked the fine-grained spatial resolution to pick up these differences.

Based on recent findings of Van der Veen et al. (2013) we anticipated finding a larger P3 component when social-evaluative feedback communicated acceptance. Indeed, anticipated social acceptance feedback elicited largest P3 amplitudes, and an overall trend was observed in the current study suggesting that the P3 was larger for acceptance than rejection feedback. However, these differences were not significant. This could be due to differences in sample size between our study (*N* = 31) and the Van der Veen et al. (2013) study (*N* = 17), and/or differences in the number of experimental trials. The current study does add an important dimension to the interpretation of the enhanced P3 after anticipated social acceptance feedback reported by Van der Veen et al. (2013). Namely, we found that an increase in SPN amplitude during anticipated social acceptance correlated significantly with P3 amplitudes in this condition. This finding is in accord with neuroimaging results reported by Gunther Moor et al. (2010b), showing enhanced vACC activity to social acceptance feedback in those individuals who also expected to be liked. These authors postulated that social acceptance is more salient when individuals also anticipate to be accepted. The current correlation between the SPN (anticipation) and P3 (feedback processing) may provide further support for this notion.

There are a few limitations to the current study. First, the limited sample size (*N* = 31) and the use of an undergraduate sample of female participants (instead of using a group comparison between healthy controls and a clinical sample) impede the generalization of the current findings to patients with social anxiety disorder. Second, no causal interferences can be made from the correlational analyses with respect to FNE or social anxiety. Moreover, given the absence of a correlation between self-reported social anxiety (LSAS) with the behavioral and electrocortical data, the current findings may only be related to a certain aspect of the social anxiety spectrum, namely social-evaluative threat. Therefore our findings are preliminary and in future studies it will be important to examine whether this pattern of findings exists in a group of clinically diagnosed socially anxious patients. A third limitation of the current experimental design is that the psychological experience of predicting to be liked or disliked perfectly covaries with the physical attributes of the feedback stimulus (i.e., the word “yes” or “no”). Since no counterbalancing was possible using these feedback stimuli, differences in SPN amplitude between conditions may partly be due to the imagination of these feedback stimuli while anticipating this type of feedback. We argue that this effect would be negligible; however, futures studies may consider using different feedback stimuli (e.g., symbols) that are presented in a counterbalanced fashion. Fourth, our participants were not asked about their subjective estimates of the relative proportion of receiving positive or negative feedback, before and after the study. This information could yield individual differences in subjective estimation of the proportion of acceptance or rejection feedback that participants received. For example, participants higher in FNE may overestimate the proportion of social rejection feedback. Although we did not find such differences based on the actual judgments during the task, Somerville et al. (2010) demonstrated that participants with high self-esteem overestimated the proportion of social acceptance feedback. Future studies should ask this information from participants in exit interviews, as this may shed light on perceptual biases in interpreting social-evaluative outcomes.

In conclusion, by investigating both behavioral and electrocortical correlates of social-evaluative processes, the current study demonstrates that individuals high in FNE display information processing biases during the anticipatory stages of social evaluation. In contrast to the prevailing notion that socially anxious individuals anticipate to be socially rejected, we did not find evidence that confirmed this bias in females with higher FNE levels. Results did show, however, that females higher in FNE needed more time to make their judgments about an upcoming social-evaluative outcome. This significant increase in RT may reflect heightened self-focused attention and vigilance imposed by the upcoming social-evaluative threat. An interesting objective for subsequent investigations is to examine whether the SPN during social feedback anticipation is driven by uncertainty and/or the intrinsic motivation to be socially accepted, and how these processes are (differentially) modulated by FNE. Taken together, this study accentuated the importance of a temporally fine-grained electrophysiological method to assess social-evaluative information processing. Results provided novel insights into the behavioral and electrocortical correlates of social-evaluative feedback anticipation that may set the stage for future studies on delineating trait markers of social anxiety.

## AUTHOR CONTRIBUTIONS

Melle J. W. Van der Molen, Michiel Westenberg, and Bregtje Gunther Moor: Conceived and designed the experiment; Eefje S. Poppelaars, Caroline T.A. Van Hartingsveldt, and Anita Harrewijn: Performed the experiments; Melle J. W. Van der Molen: Analyzed data and wrote the paper; Melle J. W. Van der Molen and Michiel Westenberg: Revised the paper.

## Conflict of Interest Statement

The authors declare that the research was conducted in the absence of any commercial or financial relationships that could be construed as a potential conflict of interest.

## Footnotes

1 The presentation of the participants’ judgments (i.e., to the left of the face) and the feedback (i.e., to the right of the face) may result in contralateral event-related brain activity. As we were only interested in the events prior to (SPN) and during feedback-processing (FRN/P3), participant’s attention to the right visual field may be associated with ERP maxima over the left hemisphere. However, this potential lateralization confound would not affect the hypothesized amplitude differences in the SPN and feedback-related conditions, as lateralization of stimuli did not vary by condition.

2 We also analyzed the SPN in an 800 ms time-window before the onset of the feedback using a mean amplitude calculation. This analysis did not yield significant differences from the 200 ms time-window analysis. As can be seen in **Figure 2**, the difference in SPN amplitude are most pronounced during the last 200 ms, indicating that the 200 ms time-window best reflects the anticipatory process (Poli et al., 2007).

3 A *post hoc* power analysis was run to determine whether our participant sample was large enough to obtain appropriate statistical power. Results of this analysis revealed that a sample of N = 37 is required to obtain an appropriate level of statistical power at 0.80. Therefore any conclusions about the significance of the results presented below were made with a certain level of caution.
